# A Systematic Review of the Application of Camera-Based Human Pose Estimation in the Field of Sport and Physical Exercise

**DOI:** 10.3390/s21185996

**Published:** 2021-09-07

**Authors:** Aritz Badiola-Bengoa, Amaia Mendez-Zorrilla

**Affiliations:** eVida Research Group, University of Deusto, 48007 Bilbao, Spain; aritz.badiola@opendeusto.es

**Keywords:** human pose estimation, sport, physical exercise, human joint estimation, keypoint detection

## Abstract

Human Pose Estimation (HPE) has received considerable attention during the past years, improving its performance thanks to the use of Deep Learning, and introducing new interesting uses, such as its application in Sport and Physical Exercise (SPE). The aim of this systematic review is to analyze the literature related to the application of HPE in SPE, the available data, methods, performance, opportunities, and challenges. One reviewer applied different inclusion and exclusion criteria, as well as quality metrics, to perform the paper filtering through the paper databases. The Association for Computing Machinery Digital Library, Web of Science, and dblp included more than 500 related papers after the initial filtering, finally resulting in 20. In addition, research was carried out regarding the publicly available data related to this topic. It can be concluded that even if related public data can be found, much more data is needed to be able to obtain good performance in different contexts. In relation with the methods of the authors, the use of general purpose systems as base, such as Openpose, combined with other methods and adaptations to the specific use case can be found. Finally, the limitations, opportunities, and challenges are presented.

## 1. Introduction

Human Pose Estimation (HPE) consists of estimating the position of different parts of the body, such as the joints in a 2D or 3D space depending on the estimation type, normally from visual information, such as images, and sometimes through other additional data obtained by different types of sensors, such as inertial sensors or depth sensors. This field of research can be considered a combination of Data Processing and Artificial Intelligence, more specifically, Computer Vision.

Since 2014, and mainly the past 5 years, the use and interest in HPE has increased, mainly due to the introduction of Deep Learning to the field [1]. The methodology has evolved from the first simple neural networks to the complex Convolutional Neural Networks (CNN) of today. The use of filters to obtain lines, edges, silhouettes, and other remarkable characteristics of the elements contained in images, as well as the capability of providing information to a system that can learn some characteristics and then detect them when a similar situation is given, have supposed an inflection point.

There are some available surveys that give an overall view on the papers as well as the State Of The Art (SOTA) systems, such as [2,3]. The first one is focused on monocular approaches, while the second survey gives an overall view of the different types of HPE systems, such as 2D and 3D, single view and multi-view, single person, and multi-person, and so on. Depending on the different characteristics of the problem, different types of systems can be found. A view of the available public datasets, as well as the used metrics, is presented as well..

Both surveys and preliminary analysis of the available papers about HPE show how the applications of HPE have increased. Different uses of these types of systems can be found, such as in the field of health [4], Human Computer Interaction (HCI) [5], Motion Capture (MoCap) systems [6], Virtual Reality (VR) [7], Augmented Reality [8], exergames [9], and so on. For some applications, the systems are based on general-purpose systems that have shown very good performance in benchmarks. In the recent literature, we can find some examples of general-purpose HPE systems, which implement innovative methods and in which different systems will be probably based, such as [10], which additionally includes the publicly available code. This system could be a very good starting point to develop a HPE system applied to Sport and Physical Exercise (SPE), as it has obtained very good results in a benchmark with images in the wild, and thus in the context of in-the-wild predictions, could be a very good option. Another good starting point for applying HPE in SPE is the system developed in [11], which is publicly available as well. This system is specialized in situations of self-contact, so, it could be a very good base for developing a HPE system applied in yoga, for instance.

This paper consists of a systematic review based on the PRISMA guidelines, in which the objective is to provide a similar analysis of the literature as provided by other HPE survey or literature review papers, but that is focused on the application of HPE to the field of SPE, highlighting some aspects related with these systems as well as applying an analysis and review that follows the criteria specified throughout the paper. The importance of the evaluation, taking into account the used metrics and data, as well as the provided information and detail of the process, is highlighted, but other aspects related to the quality of the work and the paper are considered too. The innovations and evolution of this specific field, as well as the problems and opportunities, will be presented. 

As it can be seen in the literature reviews related to general-purpose HPE systems, those systems are trained in a variety of contexts and actions, but they are not specifically focused on SPE. The movements in sport and during physical exercise tend to be different from the “standard” movements, sometimes being very explosive movements, others including occlusions of other players or tools, and others including more challenging body positions, such as in gymnastics or yoga. So, even if a general-purpose system can be applied in those contexts, depending on the sport, exercise, or specific needs, it will not perform as well as a more specialized system that is adapted to each context and trained with specific data. This is why it is important to analyze if a general-purpose system can be used in SPE, in which sports it performs better as well as getting to know the needs of adaptations to improve the performance, even if the evaluation metrics and base architectures are the same.

Several research questions are presented in Table 1, and by the literature review. The discussion section will try to give answers to these questions, as well as reach some conclusions.

The structure of this paper is as follows. Section 2 presents the evaluation methods used by the authors. The evaluation of the systems is considered one of the most important aspects of any system, as it serves as a tool to measure the performance of a system and be able to compare it with other authors’ works. The most used metrics, as well as datasets, will be analyzed, highlighting the fact that there are few 2D datasets for training HPE systems specialized for its application in the field of SPE, such as Leeds Sports Pose, Penn Action, and PoseTrack, and some others which are not specifically designed for this area, but include some content about some sports or physical activities, such as the broadly used ones as 2D HPE benchmarks, Common Objects in Context (COCO), and Max Planek Institut Informatik (MPII). Then, analyzing the availability of 3D datasets, a lack of sample amount as well as variety in terms of activities is detected, being able to find some datasets such as Demo for Martial Arts, Dancing and Sport Dataset (MADS), but still not being enough to improve specialized systems on SPE. Then, the literature review is presented, first, introducing the used methodology and criteria for the paper evaluation. Finally, the paper finishes with a discussion about the analyzed field, presenting some key ideas and conclusions, as well as giving some ideas of the possible future paths of the topic of HPE application in SPE.

## 2. Analysis of the System Evaluation Methods

As mentioned previously, before presenting the used methodology for the systematic review, it will be interesting to analyze the evaluation methods used by most authors, to understand how the performance of this type of system is evaluated. There are two key elements involved in the evaluation of an HPE system: metrics and data.

### 2.1. Metrics

The most used metricsthrough the analyzed literature are two, the first one being the evaluation metric used for the public benchmark COCO, and the second one a metric that can be used in 2D as well as 3D human pose estimation:

- Object Keypoint Similarity (**OKS**):
  ○ Commonly used in the COCO Keypoint Challenge.
  ○ It is formulated as Equation (1):
    (1) $$\mathrm{OKS}=\frac{\sum_{i}exp\left(-d_{i}^{2}/2s^{2}k_{i}^{2}{}_{\;}\right)\delta (v_{i}>0)}{\sum_{i}\delta (v_{i}>0)}$$
  ○ Where *d_i_* is the Euclidean distance between the detected keypoint and the corresponding ground truth, *v_i_* is the visibility flag of the ground truth, $\delta (v_{i}>0)$ is referring to those samples that are labeled, s is the object’s scale (square root of the object segment area), and *k_i_*s a per-keypoint constant that controls falloff.
  ○ To put it simply, OKS plays the same role that Intersection over Union plays in object detection. It is calculated from the distance between predicted points and ground truth points normalized by the scale of the person. Typically, standard average precision and recall scores are reported in papers: *AP^50^* (Average Precision at OKS = 0.50) *AP^75^*, *AP* (the mean of AP scores at 10 positions, OKS = 0.50, 0.55..., 0.90, 0.95), *AP^M^* for medium objects, *AP^L^* for large objects, and Average recall (AR) at OKS = 0.50, 0.55..., 0.90, 0.955.
- Percentage of Correct Keypoints (**PCK**): A detected joint is considered correct if the distance between the predicted and the true joint is within a certain threshold.
  ○ Some examples:
    ◾ PCKh@0.5 is when the threshold = 50% of the head bone link
    ◾ PCK@0.2 = Distance between predicted and true joint < 0.2 * torso diameter
  ○ Sometimes 150 mm is taken as the threshold.
  ○ This alleviates the shorter limb problem since shorter limbs have smaller torsos and head bone links.
  ○ PCK is used for 2D and 3D (PCK3D). Again, the higher the better.

Apart from the previously presented metrics, the use of other metrics can be seen in the works by several authors, such as Root-Mean-Square Error (RMSE) and Mean Per Joint Position Error (MPJPE) [12], or their own evaluation metrics, including some parameters from the estimation that are not normally included, such as Frames Per Second (FPS) in combination with sensitivity and precision in [13] or their own accuracy calculation methods that are explained in the papers [14,15]. Another metric used in some evaluations is Percentage of Correct Parts (PCP), which calculates the detection rate of limbs. The problem of this metric has been widely discussed by different authors, such as in [16], in which the benchmark MPII is introduced. With PCP, a limb is considered as detected if the distance between the detected limb and the ground truth limb is smaller than half of the limb length.There is a penalization in relation with short limbs, as they must be localized with higher precision. This is one of the most important reasons why most authors prefer to use PCK or PCKh, as the distance between the estimated and the ground truth joins is normalized with respect to the torso size, which makes the evaluation of the estimation equal throughout all the limbs.

Other metrics are used apart from the estimation of the joints of the body, such as in [17,18] focused on the estimation of the Center of Posture (CoP) or Center of Mass (CoM). In those cases, the error is calculated in relation to the ground truth location.

### 2.2. Data

Just like metrics, data is another key point of the evaluation of these types of systems. It is probably one of the most important elements related to the development of any deep learning system, as, apart from conforming the comparison tool between systems, the data is what gives to the system samples from which it should learn. Having good quality publicly available datasets is essential for the machine learning field; thus, taking into account its importance, a summary of the most important and high quality publicly available datasets related to the application of HPE in the field of sport are provided in Table A1 and Table A2 (all the tables of this section, Table A1, Table A2, Table A3 and Table A4, are included in Appendix A. Data). Apart from the datasets containing only content related to the field of sport, other datasets which are not developed specifically for that task can be interesting to use due to the nature of the actions present in the datasets, including SPE activities. These can be found in Table A3.

Additionally, an overall analysis of the data type and sources used by some of the most remarkable papers has been performed, providing a summary in Table A4. The papers presented in this table have been selected following the filtering method and criteria explained in the methodology section. As seen in the table, there is a big variety concerning the used data, including, for example, public overall HPE datasets, those focused specifically on SPE, and those developed using MoCap systems for a specific use case.

As it can be seen in Table A1, Table A2, Table A3 and Table A4, the first and most important conclusion that can be reached is that there is a lack of 3D HPE datasets for the development of this kind of system in the field of SPE. Only 2 high-quality public 3D HPE datasets can be found, and both are quite specialized to specific SPEs. One to martial arts and dance, and another one to football. So, there is no general sport or exercise dataset available in 3D, and there is a lack of this type of dataset for other activities different from those mentioned.

Regarding publicly available 2D HPE dataset specifically developed for its application in sport, only 2 general sports datasets have been found, which, even being more than what has been found in relation with 3D, can still be considered insufficient for the development of those systems. In this case, the images of the datasets include different types of sport and actions, which could be beneficial for the generalization of the problem in the field of sport. Apart from that, another publicly available dataset is focused on football. As for the 3D dataset, a lack of variety in specialized datasets exists, as some sports need a big data amount including use cases in which the most common problems of those cases are faced, as in yoga for rare positions, gymnastics for different body orientations, swimming for underwater conditions, and specific conditions and wearable tools of different sports.

Most of the 2D datasets are created using manual annotations, while most 3D datasets are generated using a MoCap system, such as Kinect or other more complex commercial ones. This could be the reason to have more 2D datasets available and more variety from the point of view of actions, actors, scenarios, and so on. As the process of manual annotation is easier from the technological point of view and can be applied in different contexts easily, it allows authors to work in this area in a deeper and wider way, while authors that want to work with 3D data need to use public datasets created using a MoCap system, and because of this have a limited amount of variety, or get their own MoCap system, and still have the limitations of the needed setup for its use. The only work that addresses the problem of generating ground truth for 3D HPE outside the lab environment is [19], generating the 3D HPE benchmark focused on football KTH Multiview Football Dataset.

A deeper analysis of Table A4 is provided in the results sections of this paper.

## 3. Methodology

In the following sections, the methodology that has been followed for the systematic review will be explained, including what has been analyzed, how and the sources of the papers. For this systematic review, one reviewer screened each record of the literature, the main author of this paper, being 2021/08/01 the last date when the different paper databases were consulted.

### 3.1. Inclusion and Exclusion Criteria

Three exclusion criteria have been applied for the review:

- Date: only papers from the year 2014 to 2021 have been included in the search, as 2014 is the year in which authors started to use Deep Learning for HPE tasks, so, the performance improved and its use started to increase.
- Publication type: only papers published in journals and conferences with high impact in the field of Computer Science have been included.
- Estimation type: only HPE has been considered, understood as an overall body pose estimation, as explained in the introduction. So, for example, no eye-pose estimation or hand-pose estimation has been considered during the research. In any case, only general-purpose systems have been found related to those two pose estimation systems, not specifically applied in SPE.

The inclusion criteria are explained in the following paragraphs, but, in summary, any paper from 2014 to 2021, and published in a journal or a conference of high impact, is included, and if it includes any of the terms explained in the next lines and are related with the topic of HPE applied to SPE, which includes a high variety of sports and physical exercise activities, such as running, walking and jumping, not being necessary to practice them in a competitive way.

**Figure 1:**
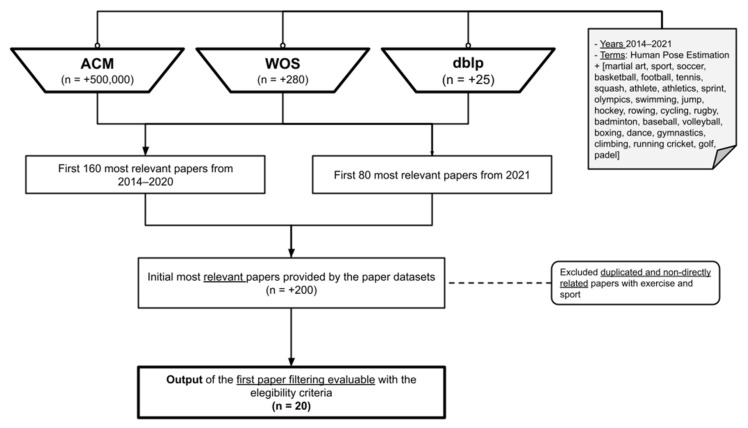
Paper selection flow diagram.

In order to search for the papers related to the topic of this literature review, the following terms were used, in conjunction with the term ‘Human Pose Estimation’: sport, martial art, soccer, basketball, football, tennis, squash, athlete, athletics, sprint, olympics, swimming, jump, hockey, rowing, cycling, rugby, badminton, baseball, volleyball, boxing, dance, gymnastics, climbing, running cricket, golf, and padel. It should be taken into account that the search took place using the terms as conjunction, so, including different terms will not limit the obtained results. The main term to be searched is ‘sport’, as it should appear in the keywords or other relevant sections of any other paper including any other term searched. Anyway, as some papers could be focused in terms such as ‘athlete’ or ‘olympics’, more than in the term ‘sport’, they have been included as an added value, including the most common sports. In total, more than 500,000 papers were obtained as results of the search in the Association for Computing Machinery Digital Library (ACM DL), more than 280 in the Web of Science (WOS), and more than 25 in dblp. In the case of ACM DL, a big part of the papers was not directly related with the topic, as they are related with general HPE or with hand or objectpost estimation, while in WOS and dblp, mostly all the papers were related with the topic. An initial filtering was applied to the first 160 most relevant papers from each paper dataset from 2014 to 2020, and the first most relevant 80 papers from 2021, removing duplicated or very similar ones, obtaining 20 papers as output. This process can be easily visualized in the flow diagram contained in Figure 1. One paper is considered to be part of the most relevant group if it fulfills the inclusion and exclusion criteria, and the results of the study are properly documented, so, if qualitative and/or quantitative results are provided, and/or if the used method is publicly available and can contribute to the work of other authors.

Other paper repositories were considered for inclusion in the research, but as most of them were not as specialized in Computer Science as ACM DL, WOS and dblp are, the number of relevant papers was not so big and the quality was not so high. For example, Pubmed was considered to be included in the research, but the focus of this search engine is on medicine, so the number of papers related to the current topic was not so high, and the perspective of the work was different, so it has was not included.

### 3.2. Quality Criteria

It is considered essential to specify how the evaluation of the papers from the first filtering was applied, to be completely transparent and as objective as possible. With this objective, a table of classification criteria (see Table 2), with their category, description, possible values, and importance in the evaluation process was created. The objective of the table is to provide a view of the most representative papers of the past years related to the application of HPE in the field of sport and physical activity. The metrics defined in Table 2 are defined by the authors attending to the content of the paper itself and other quality metrics not usually evaluated, such as replicability through the code or datasets, performance, or innovation.

The criteria were selected looking at the most important aspects of the works related to the topic, and the filtering described in Figure 1. In order to understand Table 2 properly, it is considered of interest to mention in detail some criteria: (1) all the criteria are supposed to be as objective as possible, even if there is some subjective interpretation, such as in the criterion of Innovation. There is some level of subjectivity in terms of the importance is given to each of the criteria by the weight attribute. Some criteria are binary variables, as only if the criterion is fulfilled or not is wanted to analyze, while other criteria accept 3 values or even ranges from one value to other. (2) criterion 10 only measures if the paper has any citations apart from the author’s ones. This has been established like that because taking into account the low amount of available research about this specific topic, the number of citations is very low, so, any citation, out of the self-citations, is considered a quality measure, as this indicates that the work itself, as well as the publication of the method and results during the research and development, have been of interest and useful to other works. The specificity of the work can be a handicap in terms of citations, so, considering the low amount of research in the field and this fact, we decided to evaluate the papers giving most of the importance to the research and development work, as well to the results, and give less importance to the citations of the paper.

As expressed before in the paper, the evaluation of the systems is considered very important when analyzing the work done, as it serves as a way of comparison with other systems as well as a tool to measure the quality of the work. So, as it can be seen in the table, not providing information related to the evaluation process would mean a reduction of the result of the paper evaluation of at least 3.5 points out of 11, so, applying these eligibility criteria the paper would be considered at least 30% worse.

As seen in Table 2, different variables are assessed, even if a little bit of subjectiveness can influence the analysis, those variables are objective. For example, different performance variables were taken into account when analyzing the developed systems by the authors, including accuracy and error, which, at the same time, can include different metrics, such as OKS, PCK, RMSE, and PCP. Another performance variable taken into account during the analysis was FPS, as in some cases the speed of the system can be essential for the applicability of the application in the analyzed context. Other factors that influence the analysis of the papers include how the used data is gathered, the quality of the data, the availability, and the limitations, which could be due to the low amount of samples, or the low variety of the images.

### 3.3. Information Sources

As indicated earlier, three paper databases with high-quality computer science papers were selected, which are summarized in Table 3.

## 4. Results

First, Table 4 is presented, summarizing the technical aspects of the papers, and then, the results of the application of the quality criteria are presented.

Openpose* has a multi-stage CNN architecture. The image is analyzed by a CNN (initialized by the first 10 layers of VGG-19 and fine-tuned), generating a set of feature maps that is input to the first stage. The first stage produces a set of PAFs iteratively concatenating the prediction with the original image features to produce refined predictions. The second stage predicts confidence maps, using the same iterative process of the first stage. PAFs are very useful for part association, while confidence maps are used for part detection. Each stage is composed of several convolution blocks, which, at the same time, are formed by 3 3 × 3 convolutional kernels, concatenated following an approach similar to DenseNet [50], which reduces the computation.

After the first filtering of the papers, which removed all the papers that are not directly related to HPE applied to sport and physical activity, the duplications and very similar papers were deleted. Finally, 20 papers were obtained, taking into account the previously presented eligibility criteria, to present the most interesting papers of the field. The application of the eligibility criteria can be seen in Table 5, and the topic of each paper as well as the information related to the used data for the development/evaluation of the system in Table A4.

As seen in Table 5, 9 papers get a high score of 9 or higher out of 11, 5 papers have a score between 7 and 8.9, and 6 papers obtained fewer points than 6.9, after the evaluation applying the criteria specified in Table 2. Analyzing the information provided in Table A4 and Table 5 in combination, it is possible to get an overall view of the most remarkable literature regarding the application of HPE to sport, including those papers which try to improve the current SOTA, as well as those which try to combine different methods to create new possibilities regarding specific use cases.

**Figure 2:**
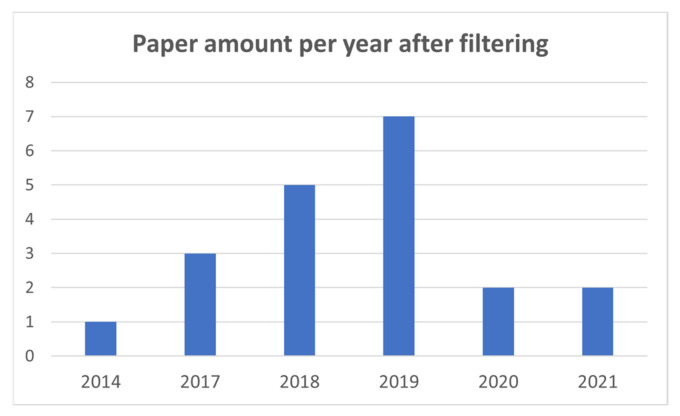
Paper amount per year after the application of the filtering process.

**Figure 3:**
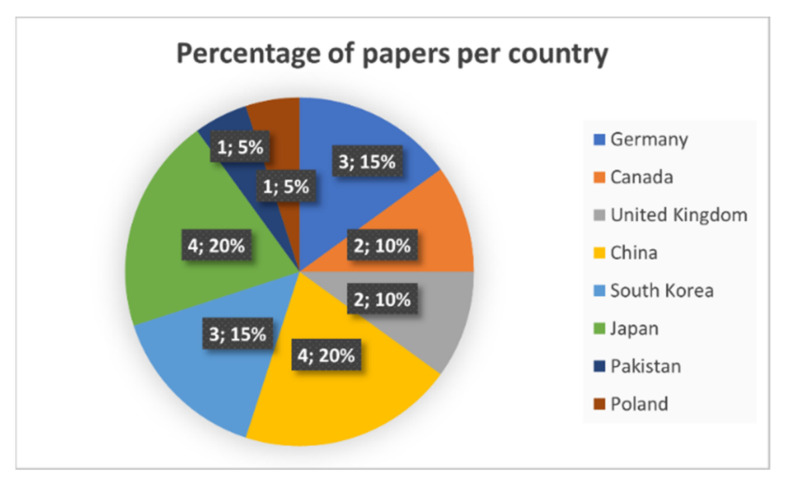
Percentage of papers per country with publications that fulfill the quality criteria successfully.

In addition, data about the years and countries of the publications is provided in Figure 2 and Figure 3. As it can be seen, after the application of the filtering, Asia is the most active continent in relation to the application of HPE to SPE, and 2018 and 2019 are considered the years with the biggest number of published papers that successfully fulfill the specified criteria in Table 2. 

**Figure 4:**
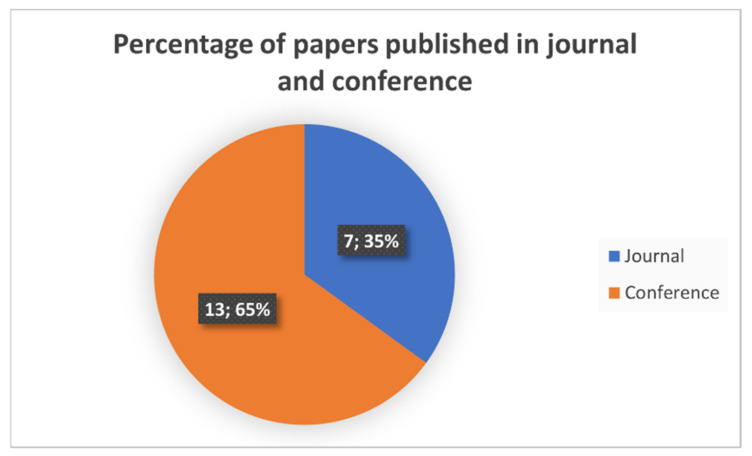
Percentage of papers published in journals and conferences that fulfill the quality criteria successfully.

It is interesting to analyze as well where the papers were published, in a journal or a conference. With this purpose, Figure 4 is presented, and as it can be seen, most of the papers were published in conferences.

In addition to the papers presented in Table A4, Table 4and Table 5, there are other papers in which different HPE methods are used in different sports with different interesting objectives. For example, we can find some authors which make use of *OpenPose* [21] for **action detection or positional predictions of different elements in the sports practice**, such as for badminton in [51], volleyball in [52], and tennis in [53]. There are other works with their own HPE implementations looking for real-time forecasting of trajectories, such as for table tennis in [54]. Other works look for specific actions when analyzing the frames of sports videos, as in [55] for athletics. Some authors are more interested in sports or physical exercise in which less “action” or movement is present, but more complexity in terms of poses is found, such as Taichi [56] and Yoga [57]. These works specifically are focused on providing the practitioners a tool to check the correctness of their poses, to learn more easily.

## 5. Discussion

In this section, the objective is to answer the questions in Table 1, as well as provide a conclusion regarding all the content presented in this literature review, and analyze the possibilities concerning the future applications and paths.

First of all, as a conclusion regarding the provided statistical information in the previous section, it can be said that taking into account the number of papers published in general about this topic, the topic of this systematic review can be considered a **hot topic**, which is attracting the interest of the research community, mainly since the year 2017.

After analyzing the review Table 5 from the previous section, and **in terms of overall form and content of the papers**, it can be concluded that concerning paper quality, implementation, use of HPE in SPE, performed evaluation, and obtained results, [46] can be considered a reference paper to replicate in terms of form. In this paper, the authors have a specific objective that is clearly presented, as well as the method they follow. They make an analysis of the needs of the specific context in which HPE is wanted to be applied, state of the art methods of general-purpose HPE are analyzed, used as examples, and adapted to the needs. This method is combined with other technologies to contribute to a specific area of SPE, and results with other methods are compared using well known metrics and taking into account other aspects of the systems apart from the accuracy, such as the speed or the real-time applicability. Publicly available benchmarks are used, which makes possible the comparison of the performance of the system with others. A dataset including images of the specific use case is developed as well and compared the obtained results with other SOTA HPE systems, which is a very good way of evaluating the developed system. The only negative aspect of the paper is related to the replicability of the work, because, even if a comparison of the developed method and other HPE systems is provided, the code is not publicly available, nor the developed dataset. Saving the work in a private way is understandable because the developed system could have future commercial use, but making public the used dataset for the evaluation and/or training should be considered an interesting approach to be able to contribute to the research community and enable others to compare their systems and contribute to the research community too.

In general, all the papers provide a good abstract and explain their experiments and evaluation properly, but, in a lot of cases, the **analysis of the limitations of the study**, or the faced problems, is missing. This can be interpreted as an intend to show only the positive aspects of the work to make it more attractive but analyzing the negative aspects and showing them can be a very good habit to improve the quality of the systems by the research community. In any case, most of the papers provide innovative solutions applicable in sport or physical exercise, with good results.

As this topic is quite specific, and, as most of the works are quite recent and there is not a big amount of research papers per year, the citations per paper are quite low. In some cases, there are not citations, but, as explained in a previous paragraph, this can be because some papers have been recently published.

Different conclusions can be reached regarding different aspects of the analyzed information during the literature review. First of all, as a general conclusion, **the lack of publications regarding the specific topic of HPE applied to SPE** can be detected. Even if hundreds of papers can be found using related terms for the search, finally, few related high-quality papers are available.

Regarding the topic of the evaluation data, the conclusions that can be reached after the analysis of its availability are:

- A bigger amount of 3D data is needed.
- A higher variety in the type of actions/sports present in 3D datasets is needed.
- The amount of 2D data could be enough for the development of a generic 2D HPE system to be applied in sports, but, when applying that system to specific sports, with their specific characteristics and problems, the error could be higher than expected from the overall sport evaluation. So, more variety of sports is needed, and a bigger amount of data per action/activity, including different challenges for the task of HPE.

**Publishing the datasets developed by each author** could be a very good way of contributing to solving this lack of publicly available data. Each contribution will be part of the data that could be used by different systems to solve the problems faced by the dataset authors or related problems of similar sports or activities.

As seen in Table A4, **most of the HPE systems applied to sport or exercise are 2D** systems, and those which are 3D systems have developed their own dataset for the specific use case, usually not making it available for the research community. This predominance of 2D systems can be due to the previously mentioned lack of 3D HPE datasets for SPE, so, there is a need for a bigger number of samples as well as an increase in the variety of activities. In addition, there are publicly available high-accuracy and fast systems such as OpenPose, introducing their method in [58], a paper that has been used by several papers to use HPE in different fields and for different applications, such as in the case of [59], in which their previous less effective player tracking system is replaced by this model to implement a squash player tracker effectively. The paper [58] has been updated and amplified in terms of detail and complexity, introducing [21], which as previously mentioned, has served already to apply HPE in different sports to different authors, and probably will continue to be used for 2D HPE problems, and maybe, would be applied to solve 3D HPE problems, by the integration to other methods to estimate the depth of the keypoints.

One of the most surprising aspects of the available literature is that a big part of the papers does not use publicly available datasets to evaluate their systems, or they do not make their developed datasets public. As explained previously, data is a key aspect in the concept of replicability of work, as well as in terms of comparison with other systems, so, not including any evaluation with a dataset that can be accessed by other authors can be considered a quite negative aspect. Another key point regarding replicability is making the code available to other authors, and the code of the analyzed papers is not available in any case. When analyzing the literature of general use HPE systems, the code of several systems can be found. In any case, it is understandable that some authors do not consider publishing their code due to potential patent or product possibilities.

Regarding the **used data for the development and testing** of the systems, on the one hand, several papers such as [12,13,14,15,17,18,20,37,38,42], developed their own datasets using manual annotations, MoCap systems, or other ground truth generation methods, but did not make them publicly available. Other papers created and published their dataset to contribute to the research community, such as [22,30]. A big number of papers use publicly available datasets, at least in the training phase of the system. Most of the public datasets used for evaluation are 2D datasets, and in some cases, other datasets such as UCF are used to provide qualitative results of the systems. In most cases, the type of data used is the same, for input image data in combination with 2D or 3D joint localizations as ground truth, and the generated data by the system are the estimated joint localizations, and in some cases some extra information related with the performance or other physical parameters of the use case.

Obviously, and as found in the case of general use HPE systems, **CNNs are the base of the methodology of most of the systems, in combination with different methods**, such as the use of heatmaps and physical constraints to reduce the error by estimating only feasible body positions. Most of the authors use approaches previously introduced by other authors, and pretrained with public datasets, as the base of their system, and then apply methods to improve the usability of those systems in specific sports or exercise movements. It is common as well to use HPE as a tool to generate new information regarding performance parameters, location of the CoM of the athlete, application of forces, etc.

Several approaches are trying to **solve specific estimation problems** in different environments, such as the ones for basketball [14], diving [24], hockey [35], etc, while others try to create a general sports use system, such as [39,41]. Taking into account the limited amount of work in specific sports, we can say that interesting research and development can be found regarding HPE and hockey. Some of the authors of [35,60,61] are involved in the three papers, starting from [60], in which the dataset HARPE is introduced, focusing the work more on action recognition than in HPE. Then, the paper [35] is published, in which results of implementing the network introduced in [60], Stacked Hourglass, in the task of HPE are presented, and compared with the newly introduced HyperStackNet. Obviously, the newer network obtained better results, as, aside from being based in the previous network, it makes use of additional information apart from the image, including the position of the center of the body as input. Finally, in the paper [61], the dataset introduced by the first paper is improved to HARPET, including temporal information. Thanks to this, without making use of any additional information apart from the image itself as input for the network, a high PCKh score is obtained, a little bit higher than the one obtained in [60]. As a negative aspect, taking into account that we are talking about the training of Deep Neural Networks, and considering that HARPET only contains 1.200 images, the amount of data used for these papers can be considered too low, and, in addition, it has not been publicly available in any of the publications. Obviously, there is a lot of work and experimentation to do in regards to HPE and its application in hockey, and more data is missing for the training of HPE in this specific task in this specific sport, but these three papers make a good job of showing some possible paths to follow.

**From the technical point of view**, considering the carried-out research, and the results presented in Table 4, it can be concluded that there is a variety in terms of HPE application in SPE. On the one hand, several papers can be found which directly apply general-purpose HPE systems for a specific sport in a specific context, trying to measure the applicability of those systems in that specific use case. On the other hand, several papers try to improve existing systems or architectures that have shown good performance in general-purpose contexts, by applying different methods focused on solving specific problems of specific contexts, which includes the type of exercise or sport, the environment, the involved tools, or the objective of the pose estimation. For example, [24], to solve the problem of self-occlusions of athletes in the air, use the mutual relations between the key nodes in the heatmap generated by each level network. Ref. [27] create a structural-aware Spatial-Temporal relation convolution module to solve a usual problem in sports videos, which is suffering from blur due to the fast movement of athletes. Ref. [30] implement a hierarchical top-down HPE method, which makes the method invariant to rotation and occlusion, two problematic situations very common in dancing. [35,42] both focus on sports that include the use of tools, one in hockey and the other one in skiing, implement methods that can learn non-body keypoints, with interesting applications for other sports as well. In [37], the authors evaluate a widely used HPE system, and see that even if being a general-purpose system does not perform badly in the case of HPE for swimmers, it can be improved. So, they implemented three methods to solve several problems related to the visually challenging environment.

Thus, it can be concluded that the need for a specialized HPE system will depend highly on the context in which it is going to be applied, as well as the objective of its application. Sometimes, using a general-purpose system could be enough to get acceptable performance, but, in other cases, with special needs/objectives or challenging characteristics, the implementation of some methods will be necessary. In any case, more experimentation is needed in this field, as the variety of contexts to apply HPE is high, and the needs differ.

In addition, we can see a very interesting method to reduce the needed amount of estimations or manual interactions when constructing a dataset in the paper [12]. This **could be especially interesting in the case of some sports or the practice of physical exercise**, and probably is the reason why the authors decide on using this use case to test their system. In a lot of sports, there are sequences in which some “body configurations” are repeated in a cyclic way, such as in the case of rowing or running. In these cases, using a method similar to the one introduced in that paper could improve the performance of the system, as well as serve as a tool that can make easier the process of human labeling of body parts.

The paper [25] obtained good results in public datasets related to sports, but, does not manage occlusions and person pose inversions properly, so, the field of application is quite limited. If its method is combined with a method to manage occlusions, and data augmentation is applied, it could get outstanding results, generating a system that could be applied in several SPE contexts.

Another aspect to be highlighted is the focus of most of the systems in obtaining a higher accuracy or lower error, while there are few systems that take into account other aspects such as the lightness of the speed of the system, such as [13]. We think this is strange from the point of view of utility in sports, as, the need for a real-time or fast system, or the need for a light model to run in a low resources hardware could be common in the field of SPE, and it looks like few authors are focusing on those aspects.

In terms of results obtained by each paper, it can be said that the use of HPE in sports and exercise activities is very beneficial, as, apart from the biomechanical aspects of the body by the pose estimation itself, different parameters and value information can be generated for the athlete, as well as for the coaches and other sports experts. The **applicability and possibilities of HPE in sport are just at the early stages, there are still several sports and applications to test and systems to be developed**. The number of sports in which HPE has not been applied, or has been barely applied, is huge, and, as previously explained, the development focused on different aspects than accuracy or low error, such as the speed or the lightness of a system, the specialized setup to a concrete problem, or the use of low-cost hardware, could be a great opportunity to study.

Taking into account the **problems faced** by different authors when applying HPE to specific sports or movements during physical exercise, apart from the interest in getting a higher accuracy in terms of low error regarding the prediction of the position of the joints, implementing methods to avoid the problems generated by occlusions could be an interesting branch of the field to research and develop. For example, in [62], in which an analysis system for rowers is pretended to be developed, an important part of the ground truth data was excluded due to occlusion problems. Another recurrent problem when applying HPE to different sports is the huge error when rare poses are present, such as in gymnastics, pole vault, swimming, dance, etc. There are some papers, such as [63], that try to lower the problem using data augmentation methods, but there is still a lot of work to do on this topic.

Looking at the **future**, there are **interesting paths to be explored and methods to be exploited**, such as the use of GANs and synthetic datasets as a way of increasing the available data to train and test systems. As an example, there are works such as [43], in which these methods are applied as a way of reducing the amount of human work and time needed, and, as a tool for data augmentation. It can be very interesting to analyze the results of these methods, applied in different sports, contexts, and integrated with other methods and with different configurations. Another interesting area of research combining HPE with other Computer Vision algorithms applied to SPE could be the analysis of the interactions and relationships between athletes and the tools and elements involved in the sports practices, such as balls, rackets… as presented in [64]. Being able to get this data, estimate the pose of athletes with considerable accuracy, as well as track ifferent elements involved in the game, and establish relationships, could be a very useful tool for the field of sport and performance analytics. In these specific papers, the experimentation and presented results are quite limited, only qualitative results are included, but further research on this area could make huge contributions to the field of SPE.

## Figures and Tables

**Table 1:** Research questions.

Question	Purpose
Do literature and public content have bases to start applying HPE in SPE?	Understanding what are the needs of HPE systems applied to SPE and if the actual general-purpose HPE research is enough to work with its application in this context.
How is HPE applied in SPE? Which are the used architectures? Which methods improved the performance in the applied context? Is using a general-purpose system enough for getting good performance or any special adaptation or aggregation of methods is needed?	Analyze how HPE applied in SPE differs from other applications and how it is applied to each context, understanding the specific needs, and whether it is necessary or not to do extra development work for improving general-purpose systems in the application context.
Can public **2D HPE data** be found in order to be applied to SPE?	Researching on the amount of data available for training and evaluating 2D HPE systems in SPE.
Can public **3D HPE** data be found to be applied to SPE?	Same purpose as the previous one, but focused on 3D systems.
Are there a higher number of papers working in **2D or 3D** HPE applied to SPE?	Knowing if most of the research has been focused on 2D or 3D systems, and why.
Can we find a **variety of sports** in which HPE has been applied?	Check in which type of sports has HPE been applied.
Do most of the authors fulfill the concept of **replicability**?	Reviewing the training and evaluation process of the authors and checking if they provide the used data as well as other resources to replicate the experimentation and be able to compare their system with others.

**Table 2:** Paper and work quality metrics.

Metric Type	Item N	Description	Value	Weight
About the content of the paper (7 points)	1	Provides in the **abstract** an informative and balanced summary of the context of the problem, what was done and what was found	(0,1) (YES/NO)	1
	2	Provides the details about the **evaluation** process of the system (used data, evaluation metric, protocol and setup)	[0–2]	2
	3	Implements one or more methods that **improve the HPE** for the problems faced in one or more sport or exercise types	[0–2]	2
	4	Give a cautious overall interpretation of **results** considering objectives, limitations, the multiplicity of analyses, results from similar studies, and other relevant evidence	[0–1]	1
	5	Discuss **limitations** of the study, considering sources of potential bias or imprecision	(0,1) (YES/NO)	1
Other Quality Metrics (4 points)	6	**Dataset** used in the research is a benchmark or it has been made publicly available	(0,1) (YES/NO)	1
	7	**Code** is publicly available	(0,0.5) (YES/NO)	0.5
	8	**Innovation**	[0–0.5]	0.5
	9	**Performance** of the system: Accuracy and error	Depending on the average and maximum results of other works in relation to the same dataset or implementation, the work will obtain the following score depending on the percentage of quality of results in which it is: between 60–70% (0.5), between 71–85% (1), and 85%+ (1.5). If it is not specified, it is in the group of results under 60%, only qualitative results are provided or the experiment is not clear (0).	1.5
	10	It has any **citation** out of the author’s self-references (at the time of writing this literature review)	(0,0.5)	0.5

**Table 3:** Paper databases/repositories.

Name	Description	Topics	Numbers
ACM DL	Research, discovery, and networking platform focused on publications about computing	Computing topics: hardware, networks, applied computing, etc.	- Pub. years: 1936–2021 (present) - Publications: 2,927,188 - Citations: 17,358,813 - Journals: +50 scholarly peer-reviewed - Conferences: +170 conferences, workshops and symposia
WOS	Website that provides access to multiple databases (online + regional) that provide comprehensive citation data for many different academic disciplines	256 disciplines, including related to Computer Science	- Pub. years: 1900–2021 (present) - Publications: +174 M - Journals: +12,000 high impact journals (total +34,586 journals) - Conferences: +220,000 conference proceedings
dblp	Computer Science bibliography website	Computer Science	- Pub. years: 1936–2021 (present) - Publications: +5.4 M - Journals: all important Computer Science journals are supposed to be included - Conferences: all important Computer Science conferences are supposed to be included

**Table 4:** Summary table of the technical implementation aspects of HPE of the filtered papers. * more details are provided in the following paragraphs.

Paper	Base Architecture/System	Methodology
[20]	Openpose * [21].	The RGB image and depth data is obtained using Kinect. Using **Openpose** the **2D pose is predicted** and **mapped with** the acquired **depth data** to generate the 3D pose. Then, the **3D pose** is used to estimate gait parameters, as explained in Table A4.
[22]	Hierarchical poselets, based on the concept of ‘poselet’ introduced in [23].	For each **poselet**, Histogram of Oriented Gradients **(HOG)** features are constructed and a linear **SVM** classifier is used for detecting the presence of each poselet. A poselet represents a specific configuration and appearance of a body part, working in this case with 20 body parts.
[12]	The framework could incorporate any part detector. In the example, spatio-temporally-linked Pictorial Structures are used to estimate the human pose.	Implementation of an algorithm for **non-sequential propagation of keyframes** to other similar frames using a Minimum Spaning Tree **(MSP)**, reducing the amount of manual interaction or pose estimations.
[24]	10-layer hourglass network cascade model.	To solve the problem of **self-occlusions of athletes in the air**, the authors used the **mutual relations between the key nodes in the heatmap** generated by each level network, to continuously optimize the key nodes of shielding, and to improve the prediction accuracy of all key nodes.
[25]	3-part CNN architecture.	The first part is formed by the first twelve layers of VGG-19 [26]. The second part takes the set of features generated by the first part and estimated the hot spot map and loss, and the third part is divided at the same time into six parts, which use the hot spot map and loss of the previous part, and the set of inputs, to estimate hot spot maps and loss, till the result.
[27]	ResNet-50.	First of all, a binary human detection module is used to detect a human, similar to R-CNN serial models [28,29]. The CNN model ResNet-50 is used to extract features from each frame of a video. **Sports videos usually suffer from blur due to the fast movement of athletes**, so, to solve this, and, at the same time improve the performance of the system, the authors created a **structural-aware Spatial-Temporal relation convolution module**. This module analyzes the spatial relation of different keypoints in each time frame, as well as the temporal relation of each keypoint among different frames. These features are concatenated to obtain the keypoints of the analyzed person.
[30]	Processing of depth data.	The authors use a Kinect camera to obtain the depth image of a person. Then, apply an initial process for human extraction: floor-removal, a 3D-connected component-labeling technique [31] to segment the objects in the original depth image and identify human objects among the segmented ones by assuming that only humans move. Then, ridge data is generated making use of a distance transform map as in [32]. Finally, the estimation is done, starting with a calibration position of the body, and applying a hierarchical top-down HPE method, which makes **the method invariant to rotation and occlusion, two things very frequent in dancing**.
[13]	The architecture is based in [21].	Takes advantage of part affinity fields (PAFs) to preserve both location and orientation information across the region of support of the limb, which improves the estimation.
[17]	OpenPose.	The authors make use of an approach based on occupancy maps to associate person detections between viewpoints [33]. To reconstruct the person in 3D, each joint detection is back-projected using the calibration of the relevant camera to produce a ray in space, and with a least-squares solution, the “intersection” of the 3D rays is solved. In this way, the authors obtain an accuracy similar to the one obtained by marker-based systems.
[14]	VGG11	A feature fusion network is constructed using a pointwise feature, global feature, and RGB feature. C3D CNN model is used as feature extractor.
[15]	Convolutional Pose Machine (CPM) [34].	The HPE method is implemented as it is to be able to estimate other parameters related to the running form, such as speed, step frequency, and swing angles.
[35]	Stacked hourglass network proposed by [36].	The HyperStackNet architecture is divided into three parts: the original stacked hourglass network, which produces the initial heatmap of 16 joint positions, the latent pose vector, which concatenates each hourglass (there are 8 hourglass modules in the original stacked hourglass network) module’s output, and finally, the modified stacked hourglass network, which takes advantage from the information provided by the previous part to, on the one hand, improves the prediction, and on the other hand, **add two more keypoints: the hockey stick**.
[37]	CPM.	One fine-tuned CPM for each of the four main swimming styles (freestyle, backstroke, butterfly, and breaststroke). **CPMs can perform very well in general-purpose context, but visually challenging footage of swimmers may still confuse the HPE systems**, due to heavy splashes, water bubbles, or refractions, producing many false estimates and problems such as complete swaps of left and right body sides and single joint outliers. So, **the authors implement three methods to improve the performance in this context**: optimization for untangling joint swaps, a novel method for robust regression to approach the problem of filtering coordinate outliers and signal noise, and data-dependent filters for fine-tunning joint coordinates.
[18]	OpenPose.	The authors obtain the 3D position of each joint obtained by OpenPose, by applying the direct linear transform to each 2D keypoint to triangulate them.
[38]	Segmentation of the participant’s silhouettes.	Image thresholding was used for segmentation, it was applied to the blue color channel of the frame due to its significant contrast between the participant’s body and the environment. Obviously, this is a method that can only be applied in contexts like the one of this use case. The model was obtained from a swimming frame that contained a complete body segment, and the joint positions could be determined by looking for the centroid of intersection between two body parts. The proposed system was limited to the swimmers who have symmetrical butterfly stroke movement, as left and right body parts are not divided.
[39]	ResNet-101 (global network) and Region-based Fully Convolutional Network (R-FCN) (for local network).	The global network, a big deep network, estimates locations of parts using the global features, which are fed into the small network, the local one, in which position-sensitive ROI pooing based on R-FCN [40] is applied to refine the predictions using local information.
[41]	Segmentation of the participant’s silhouettes.	First, the salient region detection method is used to detect the visibly noticeable regions in the image, and then, a method for foreground segmentation by skin tone detection is implemented. By these two steps, the silhouette of a person is got. Then, five basic body keypoints are detected by using the body parts model, and seven more body keypoints are detected based on the previously detected keypoints.
[42]	Mask R-CNN [29].	Other HPE methods such as CPM were used previously, but even if the performance was acceptable, the error was higher due to outliers, and ski detection was a big problem. The authors developed a new model based on Mask R-CNN, which uses a branch to detect keypoints instead of generating segmentation masks, **being able even to learn non-body keypoints, such as ski tips and ski tails, very interesting to be applied in the field of sports**, in which, sometimes, the detection of sports tools is very interesting or even necessary depending on the objective of the application of the system.
[43]	Cascaded Pyramid Networks (CPN) [44].	First, a synthetic dataset is rendered, which is converted to a synthetic realistic dataset by the use of CycleGAN [45]. Then, the initial synthetic data, in combination with the cycled-synthetic one, and mixed with COCO, is used to train CPN.
[46]	VNect [47].	VNect is used for 2D pose estimation, which is based on ResNet50 [48]. Then, a residual linear network, based in [49], is used to recover the 2D joint positions to 3D.

**Table 5:** Results of the application of the eligibility criteria to the filtered papers. (Results are out of 11, and papers are ordered by the score in descending order). * indicates that the specified paper is quite recent, less than 3 months before this research work was developed. So, the lack of citations could be due to the fact of being a recent work.

Paper	1	2	3	4	5	6	7	8	9	10	Result
[35]	1	2	2	0.5	1	1	0	0.5	1.5	0.5	**10**
[27]	1	2	2	1	0	1	0	0.5	1.5	0.5	**9.5**
[30]	1	2	2	1	0	1	0	0.5	1.5	0.5	**9.5**
[39]	1	2	2	1	1	1	0	0	1	0.5	**9.5**
[43]	1	2	2	1	1	0	0.5	0.5	1	0.5	**9.5**
[46]	1	2	2	1	1	0	0	0.5	1.5	0.5	**9.5**
[12]	1	2	2	1	0	1	0	0.5	1.5	0	9
[37]	1	2	2	0.5	1	0	0	0.5	1.5	0.5	9
[41]	1	2	2	1	0	1	0	0	1.5	0.5	9
[22]	1	2	2	1	0	1	0	0.5	0.5	0.5	8.5
[25]	0	2	2	1	1	1	0	0.5	1	0	8.5
[42]	1	2	2	0.5	0	0	0	0.5	1.5	0	7.5
[24]	1	2	2	1	0	1	0	0	0	0	7
[13]	1	2	2	0.5	0	0	0	0	1.5	0	7
[15]	1	1	1	1	0	0	0	0.5	1.5	0.5	6.5
[18]	1	2	0	0.5	1	0	0	0.5	1	0.5	6.5
[38]	1	1	2	0	0	0	0	0.5	1.5	0.5	6.5
[20]	1	1	1	0.5	0	0	0	0.25	1.5	0.5	5.75
[17]	1	2	0	1	1	0	0	0.5	0	0 *	5.5
[14]	1	2	1	1	0	0	0	0	0.5	0 *	5.5

## Data Availability

All the links and how to obtain the presented data in this paper, if it is publicly available, can be found through the referenced papers.

## Appendix A. Data

**Table A1:** 2D Evaluation Datasets.

Dataset	Size & Source	N of Joints/N of People	Summary
LSP: Leeds Sports Pose [65]	- 2000 images - 2000 people - 252 MB - Flickr	14 (x,y,visibility)/1	- **Single view**. - 1 person doing sport per image, different people and sports included. The sport of the image is annotated. - Images from Flickr with the tags: athletics, badminton, baseball, gymnastics, parkour, soccer, tennis, volleyball. - Images annotated by hand. - Images in jpg format + joint information in MATLAB data format.
Penn Action [66]	- 2326 video sequences - Different people - 3GB - From various public video repositories such as Youtube	13 (x,y,visibility)/1	- **Single view**. - 1 person per video sequence performing a sport action. - Videos annotated using VATIC annotation tool and Amazon Mechanical Turk. - 15 sports actions in total. - Images in jpg format + joint information in MATLAB data format.
KTH Multiview Football dataset II (2D part) (extended version of the original) [19]	- 5907 images - 3 different players - 236 MB - Images from a football match	14 (x,y)/1	- **Single view** (1 orthographic camera). - Filming at 25Hz and a resolution of 1920 × 1080. - 1 person per video sequence playing football on the field during a match. - The annotation of the joints is done by hand - Images in jpg format + joint information in MATLAB data format.

**Table A2:** 3D Evaluation Datasets.

Dataset	Size & Source	N of Joints/N of People	Summary
KTH Multiview Football dataset II (3D part) (extended version of the original)	- 2400 images (800 time frames captures from 3 views) - 2 different players and 2 different sequences per player - Less than 1GB - Images from a football match	14 (x,y,z)/1	- **Multiview** (3 orthographic cameras). - Filming at 25Hz and a resolution of 1920 × 1080. - 1 person per video sequence playing football on the field during a match. - 3 cameras are used to record the player from 3 different angles, the cameraman rotates the cameras to follow the player and zooms him. - The 2D annotation, as indicated in Table A1, is done by hand, and the 3D positions are reconstructed using the method described in [67]. - Images in jpg format + joints in txt format
Martial Arts, Dancing and Sports (MADS) [68]	- 30 video sequences or different people, 53,000 frames - 24GB - Own images using a MoCap system	19 (x,y,z)/1	- **Multiview** (3 cameras). - Cameras capturing at 15fps and a resolution of 1024 × 768. - 1 person per video sequence, 6 sequences per category, and 5 action categories: Tai-chi, Karate, Jazz dance, Hip-hop dance, and different sports. - Recorded in a lab. - Ground Truth was obtained using a MoCap system by Motion Analysis working at 60fps. - Video in avi format + joint information in MATLAB data format

**Table A3:** Alternative HPE Evaluation Datasets applied in SPE by several researchers.

Dataset	Size & Source	N of Joints/N of People	Summary
PoseTrack [69]	- +1356 video sequences, +46K annotated video frames - +276K body pose annotations - Raw videos from MPII	15 (x,y,visibility)/+1	- **2D** - **Single view** - Several people performing different activities in different video sequences - Videos annotated using VATIC annotation tool - Images in jpg format + joint information in MATLAB data format.
COCO: Common Objects in Context [70]	- +200,000 labeled images - 250,000 people with keypoints - Flickr	Up to 17 (x,y,visibility)/+1	- **2D** - **Single view** - The keypoints were annotated by hand by different people using the crowdsourcing marketplace Amazon Mechanical Turk and its own interface for annotating. - Dataset for multiple tasks: Object Detection, Keypoint Detection, DensePose, Stuff Segmentation, Panoptic Segmentation, and Image Captioning. - Keypoint Detection involving dataset: jpg image train/val/test datasets and annotations in json format, including the image Flickr URL, its size, number of keypoints, and the keypoints themselves in [x,y] format.
MPII: Max Planek Institut Informatik [16]	- 25,000 images - +40,000 annotated people poses - Youtube	Up to 16 (x,y,visibility) (for the test set 3D torso and head orientation and body parts occlusions included)/+1	- **2D** - **Single view** - Keypoints annotated by hand by in-house workers and using the crowdsourcing marketplace Amazon Mechanical Turk - 410 human activities - Images + annotations in MATLAB data format - Original source videos are provided

**Table A4:** Overall view of remarkable papers on the topic of HPE in SPE and the used data.

Paper	Topic	Dataset/Data Source
Estimation of Gait Parameters from 3D Pose for Elderly Care [20]	Analysis of gait parameters (i.e., cadence, step length and step duration) of elderly people using HPE.	- RGB images + depth - Output: 3D - **Own** not publicly available data of gait using Kinect.
Discriminative hierarchical part-based models for human parsing and action recognition [22]	Human body parsing and action recognition.	- RGB images - Output: 2D **UIUC** (University of Illinois Urbana-Champaign) [71], annotated by hand, and **a sports image dataset** collected from the Internet in [72] (the annotation process is not specified). Both are **publicly** available in https://vision.cs.uiuc.edu/humanparse/ (last date accessed: 6 September 2021)
Athlete pose estimation by non-sequential key-frame propagation [12]	HPE from uncalibrated unconstrained monocular TV sports footage.	- RGB images - Output: 2D - Three sequences from the publicly available dataset **HumanEva-I** (ground truth obtained using a MoCap system). - Five TV quality sports sequences with different camera angles, zoom, and motion, which are not publicly available (**own** data). Annotated by hand, the occluded parts are not included in the error calculation as they are prone to human error.
HPE of Diver Based on Improved Stacked Hourglass Model [24]	HPE of divers.	- RGB images - Output: 2D **Publicly** available datasets **MPII** and **LSP**.
Pose Estimation of Complex Human Motion [25]	HPE of “complex human motion”, including a lot of sports activities (not managing properly occlusions and character inversion)	- RGB images - Output: 2D **Publicly** available **COCO** dataset.
AI Coach: Deep HPE and Analysis for Personalized Athletic Training Assistance [27]	Development of an AI Coach using HPE to analyze the pose of the athlete and detect “bad” poses, focused on Freestyle Skiing (athlete detection and tracking, HPE, bad pose detection).	- RGB images - Output: 2D (+ “correctness” of the pose) - Tracking tested onrgf **publicly** available **VOT2018-LT** and sports video dataset from **LaSOT**. - HPE tested on **publicly** available **Penn Action** and **sub-JHMDB** (manual annotation using Amazon Mechanical Turk).
Real-time dance evaluation by markerless human pose estimation [30]	A framework that evaluates dance performance by markerless HPE, with a special focus on correct detection in full-body rotation and self-occlusion situations.	- RGB images + depth - Output: 3D **Publicly** available datasets: **EVAL** (recorded using Kinect) for accuracy, and **SMMC-10** (ground truth from PhaseSpace MoCap system) for error. **Own publicly** available **K-Pop** (true positions labeled using a marker-based MoCap system) (https://goo.gl/NoVDm4 link provided but not working at the last accessed date: 6 September 2021).
Human Pose Estimation-Based Real-Time Gait Analysis Using Convolutional Neural Network [13]	Approach that uses HPE to detect abnormalities in gait patterns with 5 possible outputs: normal, abnormal left toe, abnormal left foot, abnormal right toe, abnormal right foot.	- RGB images - Output: 2D (+ gait output category) **Own** not public dataset of RGB images of people walking in different situations using markers for the hip, knee, and ankle (no HPE data is specified as ground truth, the walking category is labeled by hand)
Can Markerless Pose Estimation Algorithms Estimate 3D Mass Centre Positions and Velocities during Linear Sprinting Activities? [17]	Test the capacity of estimating the 3D mass center positions and velocities during linear sprinting activities using 3D HPE. (in such actions in which skeleton is pushing, current HPE methods show quite high error for the objective of the paper, at least for the proposed method)	- RGB images + depth - Output: 3D **Own** not public dataset created using maker-based MoCap system Qualysis and markerless OpenPose system to record sprints.
Human Posture Recognition and Estimation Method Based on 3D Multiview Basketball Sports Dataset [14]	3D HPE using multiview basketball sports dataset.	- RGB images + depth - Output: 3D - ModelNet40 (CAD models with category label) - **Own** basketball dataset which is not publicly available (the annotation process is not indicated)
A Mobile Application for Running Form Analysis Based On Pose Estimation Technique [15]	2D HPE applied for running form analysis using a phone.	- RGB images - Output: 2D (+running performance data) **Own** not public dataset created using a motion capture system by **Vicon Motion Systems**.
HyperStackNet: A Hyper Stacked Hourglass Deep Convolutional Neural Network Architecture for Joint Player and Stick Pose Estimation in Hockey [35]	HPE in combination with stick estimation applied to hockey players.	- RGB images - Output: 2D - First half of the network was trained with the **public** dataset **MPII**. - The whole training and testing have been performed using the dataset **HARPE** (Hockey Action Recognition Pose Estimation) (from the source paper [60] it is interpreted that manual annotation has been used, but it is not expressed explicitly) from another paper, which at this moment is not publicly available.
Kinematic Pose Rectification for Performance Analysis and Retrieval in Sports [37]	HPE of athletes using the example of swimming, with images from a single camera which records inside and out the water at the same time (additionally, implements its own method of improving the estimation by inserting the swimming style by hand).	- RGB images - Output: 2D - Pretrained with **publicly** available dataset **LSP**. - - Tested on **own** dataset not publicly available of swimming videos using one camera that records the athlete inside and outside the water at the same time, annotated by a human expert.
Estimation of Center of Mass for Sports Scene Using Weighted Visual Hull [18]	Estimation of the CoM in sports using 3D HPE information as input.	- RGB images (+ output of HPE using a method from other paper) - Output: 3D position of the CoM **Own** not public data using 5 **GoPro** cameras (used to reconstruct the 3D position, no pose data is stored) and a **force plate**, being this last one element the one that gives the position of the CoM to compare with the result of the system.
Development of a markerless optical motion capture system for daily use of training in swimming [38]	Estimation of the pose and rotation and velocity of joints of swimmers, and fluid force simulation.	- RGB images - Output: 2D **Own** not public data recorded using a single static camera underwater recording the swimmer performing butterfly stroke. The segments of the body are annotated manually.
Athlete pose estimation by a global-local network [39]	HPE of athletes using a global-local approach.	- RGB images - Output: 2D **Publicly** available datasets: **LSP** for quantitative and qualitative HPE evaluation and **UCF** for qualitative evaluation, as this last dataset is used for sports action recognition, so, it does not include any joint position annotation.
Human Body Parts Estimation and Detection for Physical Sports Movements [41]	HPE for physical sports movements	- RGB images - Output: 2D **Publicly** available **KTH Multiview Football** and **UCF Sports Actions** (it is not an HPE dataset, but it is interpreted from the paper that the joints have been annotated manually for testing) datasets.
Robust Estimation of Flight Parameters for SKI Jumpers [42]	HPE and flight parameter estimation for ski jumpers during the flight phase.	- RGB images - Output: 2D **Own** not public dataset of images of different skiers in different conditions performing jumps, with joint and ski annotations. From the paper, it is interpreted that the annotation process has been manual, but it is not explicitly expressed.
Synthetic Image Translation for Football Players Pose Estimation [43]	HPE applied to football using cameras placed far from the field.	- RGB images - Output: 2D **Publicly** available **COCO** for training and comparison of results, and **own** not publicly available dataset created using four high-view and high-class wide-view cameras located far from the field.
FuturePose—Mixed Reality Martial Arts Training using Real-time 3D Human Pose Forecasting with an RGB Camera [46]	HPE applied to martial arts using a single 720p camera and combined with a pose forecasting method and VR technology.	- RGB: images - Output: 3D **Publicly** available **MPI-INF-3D** and **Human3.6M** for pre-training and validation. **Own** not publicly available dataset of martial arts practitioners and professionals doing boxing and kicking actions gathered from the Internet.
